# Changes among male and female visitors to practitioners of complementary and alternative medicine in a large adult Norwegian population from 1997 to 2008 (The HUNT studies)

**DOI:** 10.1186/1472-6882-11-61

**Published:** 2011-08-11

**Authors:** Aslak Steinsbekk, Marit B Rise, Roar Johnsen

**Affiliations:** 1 Norwegian University of Science and Technology (NTNU), Department of Public Health and General Practice, Trondheim, Norway

**Keywords:** Complementary Therapies, Norway, Utilisation, Population

## Abstract

**Background:**

The aim was to investigate changes in the prevalence and characteristics of male and female visitors to practitioners of complementary and alternative medicine (CAM) in a large adult population from 1997 to 2008.

**Methods:**

Two cross sectional adult total population health surveys from Central Norwegian (the Nord-Trøndelag Health Studies (HUNT)). In 1997 42,277 and in 2008 50,713 respondents were included. Variables included demographics (age, education, working status), lifestyle (daily smoker, did hard physical activities), health status (self-rated health status, recent complaints, chronic complaints, psychiatric complaints, a range of diseases) and health care use (visit general practitioner, chiropractor). A test of difference between the results of multivariable logistic regression models for each year, including all variables, was used to analyse changes from 1997 to 2008.

**Results:**

In 1997 9.4% (95%CI 9.1-9.6) of the population had visited a CAM practitioner in the last 12 months and this increased to 12.6% (12.3-12.9) in 2008 (p < 0.001 for difference). Prevalence of CAM use in females was almost twice as high as that in males both years. For males, the significant changes from 1997 to 2008 (p < 0.05) were an increase in odds of visiting for those under 50 years, who had a recent complaint, were widower or did hard physical activities. There was a decrease for males who had a university degree, psychiatric complaint or hay fever. For females there was an increase in the odds for those under 50 years, who had a recent complaint or chronic complaint. It was a decrease for females with reported fair global health, psychiatric complaint, hay fever or if they had visited a chiropractor.

**Conclusion:**

The increase in visits was mainly among younger people of both genders with more limited complaints. A larger proportion of the more healthy part of the population is increasing their visits to CAM practitioners.

## Background

Complementary and Alternative Medicine (CAM) are treatment modalities outside the dominant health care system. Several studies have investigated the reasons why people use these practices [1]. Issues such as control and participation, perceptions of illness, holism and natural treatments, and general philosophies of life have been found to be related to CAM use.

There are a number of studies investigating the characteristics of CAM users. Based on data from larger cross sectional surveys, being middle aged, female and having higher education is often associated with CAM use [2]. However, there are several types of CAM use; visits to practitioners like homeopaths and acupuncturists, performing CAM self practices like yoga and self treatment with CAM products like herbs [3].

To distinguish between the different types of CAM use is intuitively an important step since those visiting practitioners could differ from those using self treatment. There are some studies that have either focused on CAM visitors or identified these among all CAM users [4-9]. Another development is to investigate changes over time. There are to date only few studies that have analysed whether prevalence and characteristics change over time [6,10-14]. In USA there was an increase in total CAM use in the 1990's [13] and this has been stable on a high level in the 2000's [11,12], a trend comparable to Australia for total CAM use although there has been an increase in visitors to CAM practitioners [6]. In smaller studies in Israel, there has been a steady increase in the proportion of adults visiting a CAM practitioner from 1993 to 2007 [7] and also Ireland saw an increase in visits from 1998 to 2002 [10].

To further expand on this research, the aim was to investigate changes from 1997 to 2008 in the prevalence and characteristics of male and female visitors to practitioners of complementary and alternative medicine (CAM) in a large adult population in Norway.

## Methods

The data are from two cross sectional total population studies conducted in one county in Central Norway, the second (HUNT 2, conducted 1995-97) and third (HUNT 3, conducted 2006-08) Nord-Trøndelag Health Study (http://www.medisin.ntnu.no/hunt/). The county and its population is considered fairly representative of Norway concerning geographical, demographic and occupational structure [15], but have no larger cities and the population has an income and education level slightly below the national average. For both surveys, all residents aged 20 years or over were invited to participate by post and received the first questionnaire (Q1) attached to the invitation. This was to be returned at a screening station where a brief medical examination was conducted and a second questionnaire (Q2) to be returned by post was handed out. The HUNT studies have been approved by the Regional Committee for Medical and Health Research Ethics, Central Norway and the Norwegian Data Inspectorate.

In HUNT 2, 92,936 inhabitants were invited and a total of 65,495 (70.5%) persons participated. Of these, 41,734 (63.7%) participants answered a question on CAM use which was in Q2 and were included in the present study. In HUNT 3, 94,194 were invited, 50,827 participated (54.0%) and 50 713 (99.8%) answered a question on CAM use in Q1.

The total population in Norway is nearly 5 million and it is among the countries that have highest total expenditure on health per capita. The Norwegian health care system includes provision of health care services for all citizens based on need regardless of personal income. CAM practitioners operate outside the government-funded health care system and everyone can call themselves a CAM practitioner and treat patients.

### CAM visitor

A CAM visitor was defined as a participant who had consulted one or more CAM practitioners by answering yes to the question: "During the last 12 months, have you visited homeopath, acupuncturist, reflexologist, layer on of hands or another alternative treatment practitioner? (Yes/No)". There was a separate question about visits to chiropractors immediately before the CAM question, but as they are authorised health personnel in Norway, visit to a chiropractor was not included in CAM visits.

### Demographics

The participants' gender, age, marital status and education level were taken from public registers and a question on cohabitation. Level of education was reclassified as compulsory school, middle level education (including vocational education below university level) and university degree. Currently working were those answering yes to being in paid employment.

### Lifestyle

Participants were classified as smokers or non-smokers based on whether or not they were daily smokers of cigarettes, cigar and/or pipe. Activity level was dichotomised to doing more or less than 3 hours of hard physical activity weekly during spare time last year.

### Health status

Several measures of self reported health status were used (question with answering categories):

1. Global health: How is your health at the moment? (poor, fair, good, very good).

2. Anxiety and depression: The Hospital Anxiety and Depression Scale (HADS-T) was used (14 items, score ranging from 0 to 42, the cut off point for the detection of any mental disorder is found to be 17 [16].

3. Recent complaint (yes to one or more of these questions):

- Have you suffered from Nausea/Heartburn/Diarrhoea/Constipation/Breathlessness in the last 12 months? (Never = No/Sometimes = Yes/Often = Yes).

- Have you experienced any stiffness or pain in your muscles or joints that has lasted for more than three consecutive months during the last year? (Yes/No).

- Have you suffered from headache in the last 12 months (Yes/No).

4. Chronic complaint: Do you suffer from any long standing (for at least one year) limiting somatic or psychiatric illness, disease or disability? (Yes/No).

5. Psychiatric complaint: Do you have or have you had psychiatric complaints that you have sought help for? (Yes/No).

6.- 8. Asthma, Diabetes, or Cancer: Do you have or have you had asthma/diabetes/cancer? (Yes/No).

9. Hay fever: Do you have hay fever? (Yes/no).

10. Cardiovascular disease (yes to one or more of these questions): Do you have or have you had Acute myocardial infarction/Angina pectoris/Stroke? (Yes/No).

11. Musculoskeletal disease (yes to one or more of these questions): Have you been diagnosed with Osteoporosis/Fibromyalgia/Arthritis/Artroses/Bechterew/Other longstanding musculoskeletal disease? (Yes/No).

### Health care utilisation

Health care utilisation was answering yes to questions about visits the last 12 months:

- Physician: During the past 12 months, have you visited a General practitioner/Specialist outside hospital/Psychiatric specialist in hospital/Somatic specialist in hospital? (Yes/No).

- Chiropractor: During the last 12 months, have you visited a chiropractor? (Yes/No).

### Statistical Analyses

Pearson chi-square tests were used to compare users with non-users for each year separately. Spearman's rho was used to check for co-linearity and the correlation coefficient was highest both years for currently working/age (1997; 0.410, 2008; 0.561) and chronic complaint/global health (1997; 0.409, 2008; 0.507). The multivariable analysis (adjusted odds ratio - adjOR) was undertaken by logistic regression using models where all variables were included to identify associations between CAM use and the other variables also for both years. Due to the large size of the dataset and the number of comparisons, statistical significance was accepted at the 1% level (p < 0.01) for these tests. To compare the findings of the bivariable and multivariable analysis for each year with each other, a test of difference for comparison of two independent studies was used [17]. For the multivariable analysis, the Ratio Odds Ratio (ROR) was calculated, which is the ration from a comparison of two odds ratios. A ROR higher than 1 indicates higher odds of visiting a CAM practitioner in 2008 than in 1997. Statistical significance accepted at the 5% level (p < 0.05) for these tests. The adjOR and ROR are given with 95% confidence intervals (95%CI). All data were analysed using SPSS statistics version 17.0.0 released Aug 23. 2008 (SPSS Inc, Chicago, USA).

## Results

The final data from 1997 comprised 19,490 males and 22,244 females and 22,998 males and 27,715 females were included in 2008.

In total 9.4% (95%CI 9.1-9.6, females 12.2%, males 6.1%) of the population visited a CAM practitioner in 1997 and this increased significantly (p < 0.001) to 12.6% (95%CI 12.3-12.9, females 16.2%, males 8.3%) in 2008 (table 1). Thus, nearly twice as many females had visited a CAM practitioner both in 1997 and 2008 compared to males. There was also a significant (p < 0.001) increase in CAM visits for both genders (males 2.2% points, females 3.9% points) from 1997 to 2008.

**Table 1:** Prevalence of visits to complementary and alternative medicine (CAM) practitioners during the last 12 months in 1997 and 2008 for males and females

Visited CAM practitioner in	Total	Male	Female
1997	(N = 41 734) 9.4% (9.1-9.6)	(N = 19 490) 6.1% (5.8-6.4)	(N = 22 244) 12.2% (11.8-12.7)
2008	(N = 50 713) 12.6% (12.3-12.9)	(N = 22 998) 8.3% (7.9-8.6)	(N = 27 715) 16.2% (15.7-16.6)

The bivariate analysis showed that for both males and females, having poorer self reported global health, higher anxiety and depression score (HADS-T), chronic or psychiatric complaint and having visited a chiropractor were the variables most strongly associated with visit to a CAM practitioner (table 2). In 2008, compared to 1997, there was a significant increase in the proportion of visitors for most of the variables. For both males and females, the most striking changes beyond the general increase were an increase in the younger age groups and among those who did hard physical activity.

**Table 2:** Bivariate analysis with percentage of visits to CAM practitioners (%CAM) in 1997 and 2008 for males and females (N given in table)

	Male						Female					
	2008		1997		2008 vs 1997		2008		1997		2008 vs 1997	
	N	%CAM	N	%CAM	Diff	P-value	N	%CAM	N	%CAM	Diff	P-value
Visited a CAM practitioner	22998	8.3%*	19490	6.1%*	2.2%*	< 0.001	27715	16.2%*	22244	12.2%*	3.9%*	< 0.001
Age group - Under 30	1852	7.9%*	2766	4.0%*	4.0%*	< 0.001	2634	13.7%*	3546	9.2%*	4.6%*	< 0.001
- 30-39	2843	9.4%*	3738	5.6%*	3.8%*	< 0.001	3998	18.1%*	4606	12.1%*	5.9%*	< 0.001
- 40-49	4546	9.7%*	4347	5.9%*	3.8%*	< 0.001	5434	18.5%*	4898	13.0%*	5.6%*	< 0.001
- 50-59	5400	8.8%*	3476	6.3%*	2.5%*	< 0.001	5989	18.3%*	3701	14.9%*	3.4%*	< 0.001
- 60-69	4650	7.4%*	2431	7.5%*	-0.1%	0.892	5106	14.8%*	2560	13.5%*	1.3%	0.117
- 70-79	2649	6.3%*	2146	7.2%*	-1.0%	0.188	3076	12.6%*	2148	10.6%*	2.0%*	0.025
- Over 80	980	5.6%*	586	9.7%*	-4.1%*	0.002	1417	9.2%*	785	9.8%*	-0.6%	0.625
Education - Compulsory school	4458	7.6%*	5435	6.3%	1.3%*	0.009	6166	14.1%*	7436	12.3%	1.8%*	0.002
- Middle level	13200	8.9%*	9336	6.0%	3.0%*	< 0.001	13216	17.6%*	9058	12.7%	4.9%*	< 0.001
- University	5067	7.0%*	4115	5.8%	1.2%*	0.016	8006	15.5%*	4886	11.3%	4.2%*	< 0.001
Marital status - Married/cohabiting	17380	8.5%	15186	6.2%*	2.3%*	< 0.001	20123	16.7%*	17107	12.5%*	4.2%*	< 0.001
- Single	4017	7.5%	3142	4.7%*	2.8%*	< 0.001	3465	15.2%*	2125	10.5%*	4.7%*	< 0.001
- Divorced/separated	1069	8.1%	615	9.4%*	-1.3%	0.363	1603	18.3%*	878	15.3%*	3.0%	0.057
- Widow(er)	461	8.7%	512	8.0%*	0.7%	0.706	2412	11.9%*	2072	10.6%*	1.4%	0.148
Currently working	15268	8.6%	13914	5.5%*	3.0%*	< 0.001	17090	17.4%*	14037	12.3%	5.1%*	< 0.001
Current lifestyle: - Daily smoker	3919	7.1%*	5295	5.1%*	2.0%*	< 0.001	5565	16.0%	6361	11.5%	4.5%*	< 0.001
- Hard physical activity	3163	9.8%*	2568	5.1%	4.7%*	< 0.001	2936	17.7%	1287	12.0%	5.7%*	< 0.001
Global Health: - Very good	3527	5.4%*	3399	3.1%*	2.3%*	< 0.001	4172	10.3%*	3864	6.4%*	3.9%*	< 0.001
- Good	13475	7.4%*	11763	5.0%*	2.3%*	< 0.001	14787	15.2%*	12524	10.4%*	4.8%*	< 0.001
- Fair	4874	12.5%*	3934	10.8%*	1.6%*	0.017	7142	21.3%*	5343	19.8%*	1.5%*	0.035
- Poor	314	17.2%*	294	18.7%*	-1.5%	0.628	389	27.8%*	332	26.5%*	1.3%	0.705
Anxiety and depression - 0-4	6196	7.6%*	6735	4.9%*	2.7%*	< 0.001	7714	13.9%*	7561	8.8%*	5.1%*	< 0.001
- 5-9	6218	8.1%*	6998	5.3%*	2.8%*	< 0.001	7351	16.5%*	7538	11.7%*	4.8%*	< 0.001
- 10-14	3066	9.3%*	3481	7.4%*	1.9%*	0.005	3930	18.9%*	3881	15.0%*	4.0%*	< 0.001
- 15-19	1123	11.6%*	1206	9.5%*	2.0%	0.109	1719	22.4%*	1646	19.0%*	3.4%*	0.014
- 20 and higher	453	11.7%*	558	13.3%*	-1.6%	0.456	811	22.3%*	872	21.1%*	1.2%	0.545
Recent complaint	15562	9.3%*	12053	6.5%*	2.8%*	< 0.001	20995	17.6%*	14642	12.9%*	4.7%*	< 0.001
Chronic complaint	7096	12.1%*	4366	10.5%*	1.6%*	0.009	8821	22.2%*	4608	19.0%*	3.2%*	< 0.001
Psychiatric complaint	2178	12.3%*	1511	13.6%*	-1.3%	0.259	4438	21.9%*	3025	20.2%*	1.7%	0.070
Diseases - Asthma	2016	9.5%	1662	8.0%*	1.5%	0.117	2706	19.0%*	1840	16.7%*	2.3%*	0.044
- Hay fever	4185	9.4%*	3197	8.1%*	1.3%	0.051	6185	19.3%*	4405	16.3%*	3.1%*	< 0.001
- Heart disease	3251	7.4%	1568	7.6%*	-0.2%	0.827	2212	14.0%*	972	13.7%	0.3%	0.830
- Cancer	1116	8.5%	558	10.0%*	-1.5%	0.305	1541	15.5%	833	13.9%	1.6%	0.302
- Diabetes	1136	8.3%	499	7.0%	1.3%	0.384	1047	13.9%	497	12.5%	1.5%	0.429
- Musculoskeletal disease	3088	11.2%*	1904	9.3%*	1.9%*	0.032	7369	19.0%*	3259	17.8%*	1.2%	0.148
Visited last year: - Physician	17769	9.5%*	11738	8.1%*	1.4%*	< 0.001	23907	17.2%*	16787	14.2%*	3.1%*	< 0.001
- Chiropractor	1980	19.3%*	793	19.2%*	0.1%	0.940	2016	29.3%*	649	34.4%*	-5.1%*	0.014

Test of proportions is used to compare changes from 1997 to 2008 with a positive difference (Diff) indicating higher prevalence in 2008.

* p-value < 0.01 for each year and p-value < 0.05 for comparison

### CAM visit males

The multivariable logistic regression showed that for males in 1997, age had no influence (table 3). In 2008 (p < 0.01) being above 50 years old (adjusted Odds Ratio (adjOR) from 0.7 for those 50-59 years going down to 0.4 for those 70 years and older) was associated with decreased odds of visiting a CAM practitioner and this was significantly lower than in 1997 (Ratio Odds Ratio (ROR) 0.4 for those 50-59 years to 0.2 for those 80 years and older).. Having poorer self reported global health was associated with increased odds of visiting a CAM practitioner both in 1997 (adjOR from 1.4 for good health increasing to 3.8 for poor health) and in 2008 (adjOR from 1.3 for good health increasing to 2.9 for poor health), and although the odds was lower in 2008, it was not statistically significantly different from 1997. Visit to a physician (adjOR 2.0) or a chiropractor (adjOR 3.2) was also associated with CAM visits in 1997 and 2008 (physician adjOR 1.8, chiropractor adjOR 2.6), but there were no statistical significant change during these eleven years. There was a significant increase (ROR 2.0) in the odds of having a recent complaint in 2008 compared to 1997, and having a recent complaint was associated with increased odds of CAM visits in 2008 (adjOR 1.9).

**Table 3:** Adjusted odds ratio (adjOR) with 95% confidence intervals (95%CI) from multivariate logistic regression models for visits to CAM practitioners for males in 2008 (N = 16311) and in 1997 (N = 16488), for females in 2008 (N = 20469) and in 1997 (N = 17587), and test of difference between the time points for males and females with Ratio Odds Ratio (ROR)

	Male ^a^						Female ^a^					
	2008		1997		2008 vs 1997		2008		1997		2008 vs 1997	
	AdjOR (95% CI)	P-value 1)	AdjOR (95% CI)	P-value 1)	ROR (95%CI)	P-value 2)	AdjOR (95% CI)	P-value 1)	AdjOR (95% CI)	P-value 1)	ROR (95%CI)	P-value 2)
Age group - Under 30	Ref		Ref				Ref		Ref			
- 30-39	1.0 (0.7-1.3)	0.912	1.2 (0.9-1.5)	0.204	0.8 (0.6-1.2)	0.359	1.2 (1.0-1.4)	0.068	1.3 (1.1-1.5)*	0.003	0.9 (0.7-1.2)	0.515
- 40-49	0.8 (0.6-1.1)	0.248	1.1 (0.9-1.5)	0.367	0.8 (0.5-1.1)	0.145	1.2 (1.0-1.4)	0.055	1.3 (1.1-1.5)*	0.003	0.9 (0.7-1.2)	0.495
- 50-59	0.7 (0.5-0.9)*	0.003	1.1 (0.8-1.4)	0.695	0.6 (0.4-0.9)*	0.017	1.0 (0.9-1.2)	0.655	1.3 (1.1-1.6)*	0.001	0.8 (0.6-1.0)*	0.044
- 60-69	0.5 (0.4-0.7)*	< 0.001	1.3 (1.0-1.7)	0.092	0.4 (0.3-0.6)*	< 0.001	0.9 (0.7-1.0)	0.091	1.3 (1.0-1.6)	0.022	0.7 (0.5-0.9)*	0.005
- 70-79	0.4 (0.3-0.6)*	< 0.001	1.2 (0.9-1.7)	0.224	0.4 (0.2-0.6)*	< 0.001	0.8 (0.6-1.0)	0.040	1.0 (0.8-1.3)	0.980	0.8 (0.6-1.1)	0.167
- Over 80	0.4 (0.2-0.6)*	< 0.001	1.5 (1.0-2.4)	0.063	0.2 (0.1-0.5)*	< 0.001	0.5 (0.4-0.7)*	< 0.001	0.9 (0.7-1.3)	0.696	0.6 (0.4-0.9)*	0.026
Education - Compulsory school	Ref		Ref				Ref		Ref			
- Middle level	1.0 (0.9-1.2)	0.778	1.2 (1.0-1.4)	0.065	0.9 (0.7-1.1)	0.263	1.2 (1.1-1.4)*	< 0.001	1.1 (1.0-1.3)	0.023	1.1 (0.9-1.2)	0.401
- University	0.8 (0.7-1.0)	0.040	1.1 (0.9-1.4)	0.154	0.7 (0.5-0.9)*	0.014	1.1 (0.9-1.2)	0.320	1.0 (0.9-1.2)	0.656	1.0 (0.9-1.2)	0.740
Marital status - Married/cohabiting	Ref		Ref				Ref		Ref			
- Single	0.7 (0.6-0.9)*	0.001	0.9 (0.7-1.1)	0.274	0.8 (0.6-1.1)	0.110	1.0 (0.8-1.1)	0.642	1.0 (0.8-1.2)	0.829	1.0 (0.8-1.2)	0.884
- Divorced/separated	0.9 (0.7-1.3)	0.684	1.4 (1.0-1.9)	0.033	0.7 (0.4-1.0)	0.075	1.0 (0.9-1.2)	0.807	1.1 (0.9-1.3)	0.551	1.0 (0.7-1.3)	0.756
- Widow(er)	1.7 (1.1-2.6)*	0.009	1.0 (0.6-1.4)	0.819	1.8 (1.0-3.2)*	0.048	1.1 (0.9-1.3)	0.523	0.9 (0.7-1.1)	0.231	1.2 (0.9-1.6)	0.187
Currently working	1.0 (0.8-1.2)	0.886	1.0 (0.8-1.2)	0.983	1.0 (0.8-1.3)	0.935	1.2 (1.1-1.4)*	< 0.001	1.1 (1.0-1.3)	0.053	1.1 (0.9-1.3)	0.296
Current lifestyle: - Daily smoker	0.7 (0.6-0.9)*	< 0.001	0.7 (0.6-0.8)*	< 0.001	1.0 (0.8-1.3)	0.816	0.9 (0.8-0.9)*	0.001	0.8 (0.7-0.8)*	< 0.001	1.1 (1.0-1.3)	0.090
- Hard physical activity	1.3 (1.1-1.5)*	< 0.001	0.9 (0.8-1.1)	0.547	1.4 (1.1-1.8)*	0.011	1.1 (1.0-1.2)	0.084	1.1 (0.9-1.3)	0.288	1.0 (0.8-1.2)	0.985
Global Health: - Very good	Ref		Ref				Ref		Ref			
- Good	1.3 (1.1-1.6)*	0.009	1.4 (1.1-1.8)*	0.002	0.9 (0.7-1.2)	0.570	1.4 (1.2-1.6)*	< 0.001	1.5 (1.3-1.8)*	< 0.001	0.9 (0.7-1.1)	0.340
- Fair	2.0 (1.6-2.6)*	< 0.001	2.4 (1.8-3.1)*	< 0.001	0.8 (0.6-1.2)	0.379	1.8 (1.5-2.1)*	< 0.001	2.5 (2.1-3.0)*	< 0.001	0.7 (0.5-0.9)*	0.003
- Poor	2.9 (1.9-4.5)*	< 0.001	3.8 (2.5-5.9)*	< 0.001	0.8 (0.4-1.4)	0.382	2.5 (1.8-3.4)*	< 0.001	3.5 (2.5-4.9)*	< 0.001	0.7 (0.4-1.1)	0.139
Anxiety and depression - 0-4	Ref		Ref				Ref		Ref			
- 5-9	0.9 (0.8-1.1)	0.322	0.9 (0.8-1.1)	0.409	1.0 (0.8-1.2)	0.981	1.1 (1.0-1.2)	0.017	1.1 (1.0-1.3)	0.020	1.0 (0.8-1.1)	0.821
- 10-14	1.0 (0.8-1.1)	0.619	1.1 (0.9-1.3)	0.511	0.9 (0.7-1.2)	0.412	1.2 (1.1-1.4)*	< 0.001	1.3 (1.1-1.5)*	< 0.001	0.9 (0.8-1.1)	0.482
- 15-19	1.0 (0.8-1.3)	0.908	1.1 (0.9-1.5)	0.271	0.9 (0.6-1.2)	0.465	1.3 (1.2-1.5)*	< 0.001	1.5 (1.2-1.7)*	< 0.001	0.9 (0.7-1.1)	0.376
- 20 and higher	0.8 (0.6-1.1)	0.198	1.2 (0.9-1.6)	0.279	0.7 (0.4-1.1)	0.093	1.1 (0.9-1.4)	0.215	1.3 (1.1-1.6)*	0.009	0.9 (0.6-1.1)	0.295
Recent complaint	1.9 (1.5-2.3)*	< 0.001	0.9 (0.8-1.1)	0.267	2.0 (1.6-2.6)*	< 0.001	1.8 (1.5-2.1)*	< 0.001	1.0 (0.9-1.1)	0.787	1.8 (1.4-2.1)*	< 0.001
Chronic complaint	1.4 (1.2-1.6)*	< 0.001	1.3 (1.1-1.6)*	< 0.001	1.0 (0.8-1.3)	0.698	1.4 (1.2-1.5)*	< 0.001	1.2 (1.0-1.3)*	0.010	1.2 (1.0-1.3)*	0.043
Psychiatric complaint	1.3 (1.1-1.5)	0.011	1.9 (1.6-2.3)*	< 0.001	0.7 (0.5-0.9)*	0.002	1.2 (1.0-1.3)*	0.005	1.4 (1.2-1.5)*	< 0.001	0.8 (0.7-1.0)*	0.032
Diseases - Asthma	0.8 (0.7-1.0)	0.048	0.9 (0.8-1.2)	0.501	0.9 (0.6-1.2)	0.368	1.0 (0.9-1.1)	0.834	1.1 (0.9-1.2)	0.431	0.9 (0.8-1.1)	0.462
- Hay fever	1.0 (0.9-1.2)	0.706	1.3 (1.1-1.6)*	< 0.001	0.8 (0.6-1.0)*	0.015	1.1 (1.0-1.2)	0.017	1.3 (1.2-1.4)*	< 0.001	0.9 (0.7-1.0)*	0.019
- Heart disease	0.7 (0.6-0.9)*	0.002	0.7 (0.5-0.9)*	0.001	1.1 (0.8-1.5)	0.496	0.9 (0.7-1.0)	0.072	0.9 (0.7-1.1)	0.366	1.0 (0.7-1.3)	0.805
- Cancer	1.1 (0.8-1.4)	0.570	1.0 (0.8-1.2)	0.867	1.1 (0.8-1.5)	0.578	1.0 (0.8-1.2)	0.974	1.2 (1.0-1.3)	0.018	0.9 (0.7-1.1)	0.163
- Diabetes	1.0 (0.7-1.2)	0.720	1.2 (0.9-1.7)	0.168	0.8 (0.5-1.1)	0.196	0.9 (0.7-1.1)	0.160	1.0 (0.8-1.2)	0.832	0.9 (0.7-1.2)	0.419
- Musculoskeletal disease	1.1 (1.0-1.3)	0.118	0.8 (0.5-1.1)	0.175	1.5 (1.0-2.2)	0.065	1.1 (1.0-1.2)	0.033	0.8 (0.6-1.1)	0.168	1.4 (1.0-1.9)	0.050
Visited last year: - Physician	1.8 (1.5-2.2)*	< 0.001	2.0 (1.7-2.4)*	< 0.001	0.9 (0.7-1.1)	0.342	1.6 (1.4-1.9)*	< 0.001	1.9 (1.7-2.1)*	< 0.001	0.9 (0.7-1.0)	0.119
- Chiropractor	2.6 (2.2-3.0)*	< 0.001	3.2 (2.6-3.9)*	< 0.001	0.8 (0.6-1.0)	0.096	1.8 (1.6-2.1)*	< 0.001	3.2 (2.7-3.8)*	< 0.001	0.6 (0.5-0.7)*	< 0.001

A ROR above 1 indicates that the adjOR for 2008 is higher than for 1997.

* p-value < 0.01 for each year and p-value < 0.05 for comparison

a all variables are adjusted for all other variables in the model.

Having a university degree was associated with decreased odds of males visiting a CAM practitioner when comparing 2008 to 1997 (ROR 0.7) although not significantly associated with CAM visits in any year. In 1997 there was no association with marital status and CAM visits, while in 2008 Single males had decreased (adjOR 0.7) and widowers had increased (adjOR 1.7) odds of CAM visits and the only significant change from 1997 to 2008 was for widowers (ROR 1.8). Both in 1997 and 2008, being a smoker was associated with similar decreased odds of CAM visits (adjOR 0.7) while doing hard physical activity was associated with increased odds in 2008 (adjOR 1.3), a statistical significant increase from 1997 (ROR 1.4). Having a chronic complaint was associated with increased odds of CAM visits in both years (1997 adjOR 1.3, 2008 adjOR 1.4), while having psychiatric complaint was only associated with increased odds in 1997 (adjOR 1.9) with a reduction from 1997 to 2008 (ROR 0.7). In both 1997 and 2008 having a heart disease was associated with similar decreased odds of visits to CAM practitioners (adjOR 0.7), while having hay fever was associated with increased odds in 1997 (adjOR 1.3) but reduced between 1997 and 2008 (ROR 0.8).

### CAM visits females

For females, having poorer self reported global health was associated with increased odds of CAM visits both in 1997 (adjOR from 1.5 for good health increasing to 3.5 for poor health) and in 2008 (adjOR from 1.4 for good health increasing to 2.5 for poor health), and although the odds was lower in 2008 it was only significantly different from 1997 for those with fair global health (ROR 0.7) (table 3). In 1997 being between 30 and 59 years was associated with increased odds (adjOR 1.3) while being above 80 years old was associated with decreased odds of visiting a CAM practitioner in 2008 (adjOR 0.8). There was a decrease from 1997 to 2008 for females aged 50 to 69 (ROR 0.8 to 0.7) and above 80 years (ROR 0.6). Visit to a physician (adjOR 1.9) or a chiropractor (adjOR 3.2) was also associated with CAM visits in 1997 and in 2008 (physician adjOR 1.6, chiropractor adjOR 1.8), with a significantly decrease from 1997 to 2008 for visits to chiropractor (ROR 0.6). Having a recent complaint was only associated with CAM visits in 2008 (adjOR 1.8), an increase from 1997 (ROR 1.8).

In 2008 (adjOR1.2), having middle level education was associated with increased odds of visiting a CAM practitioner. There was no association between marital status and CAM visits for females. Being a smoker was associated with similar decreased odds for CAM visits (1997 adjOR 0.8, 2008 adjOR 0.9). Increased anxiety and depression score (HADS-T) was associated with CAM visits both in 1997 (adj OR 1.1 for those with a score of 10-14 and above 20 to adj OR 1.5 for those with a score of 15-19) and 2008 (adj OR1.1 for those with a score of 10-14 to 1.3 for those with a score of 15-19). Having a chronic complaint (adjOR 1.2 and 1.4) or psychiatric complaint (adjOR 1.4 and 1.2) was associated with increased odds of CAM visits in 1997 and 2008, with a reduction for psychiatric complaint from 1997 to 2008 (ROR 0.7) and an increase for chronic complaint (ROR 1.2). In 1997 (adjOR 1.3), having hay fever was associated with increased odds of CAM visits, a decrease from 1997 to 2008 (ROR 0.9).

### CAM visits in 2008; males vs. females

There were most similarities among the characteristics of males and females CAM visitors in 2008 (table 4). Males had significantly (p < 0.05) decreased odds of visiting a CAM practitioner compared to females if they were 50 years and older (ROR between 0.6 to 0.5 for those 50 to 79 years old), had university education (ROR 0.8), were single (ROR 0.7), had higher anxiety and depression score (HADS-T, from ROR 0.8 for those with a score of 5 to 14 to to ROR 0.6 for those with a score above 20) and hay fever (ROR 0.8). Widowers (ROR 1.6) and males with chronic complaint (ROR 1.2) had increased odds of visiting a CAM practitioner compared to females.

**Table 4:** Test of difference between males and females in 2008 with Ratio Odds Ratio (ROR)

	Male vs. female 2008	
	ROR (95%CI)	P-value 2)
Age group - Under 30	Ref	
- 30-39	0.9 (0.6-1.2)	0.484
- 40-49	0.8 (0.6-1.1)	0.202
- 50-59	0.6 (0.4-0.8)**	0.001
- 60-69	0.5 (0.4-0.7)**	< 0.001
- 70-79	0.6 (0.4-0.9)*	0.019
- Over 80	0.6 (0.3-1.0)	0.058
Education - Compulsory school	Ref	
- Middle level	0.9 (0.7-1.1)	0.225
- University	0.8 (0.6-1.0)*	0.038
Marital status - Married/cohabiting	Ref	
- Single	0.7 (0.6-0.9)*	0.014
- Divorced/separated	0.9 (0.6-1.3)	0.636
- Widow(er)	1.6 (1.0-2.5)*	0.032
Currently working	0.9 (0.7-1.1)	0.353
Current lifestyle: - Daily smoker	1.0 (0.8-1.2)	0.932
- Hard physical activity	1.1 (0.9-1.5)	0.279
Global Health: - Very good	Ref	
- Good	1.0 (0.8-1.3)	0.814
- Fair	0.9 (0.7-1.3)	0.664
- Poor	1.0 (0.5-1.8)	0.940
Anxiety and depression - 0-4	Ref	
- 5-9	0.8 (0.7-1.0)*	0.024
- 10-14	0.8 (0.6-0.9)*	0.012
- 15-19	0.7 (0.5-1.0)*	0.024
- 20 and higher	0.6 (0.4-0.9)*	0.012
Recent complaint	1.2 (0.9-1.6)	0.182
Chronic complaint	1.2 (1.0-1.5)*	0.048
Psychiatric complaint	0.9 (0.7-1.1)	0.178
Diseases - Asthma	0.8 (0.6-1.0)	0.080
- Hayfever	0.8 (0.7-1.0)*	0.030
- Heart disease	0.9 (0.6-1.2)	0.394
- Cancer	0.9 (0.7-1.2)	0.559
- Diabetes	1.0 (0.7-1.5)	0.900
- Muscskelskeletal disease	1.4 (0.9-2.1)	0.106
Visited last year: - Physician	1.0 (0.8-1.3)	0.997
- Chiropractor	0.8 (0.7-1.1)	0.172

The test is based on the values given in table 2. A ROR above 1 indicates that the Adjusted OR for male is higher than for female.

* p-value < 0.05

## Discussion

From 1997 to 2008 there was a significant increase in the percentages of males and females visiting a CAM practitioner. For males, the significant changes were an increase in odds of visiting for those under 50 years, who had a recent complaint, were widower or did hard physical activities. There was a decrease for males who had a university degree, psychiatric complaint or hay fever. For females there was an increase in the odds for those under 50 years, who had a recent complaint or chronic complaint. It was a decrease for those with reported fair global health, psychiatric complaint, hay fever or if they had visited a chiropractor.

The main strength of this study is that it is the largest study to date comparing changes in characteristics of visitors to CAM practitioners. This allowed for both separate analysis for males and females and the analysis of a comprehensive set of explanatory variables. One major limitation was that it was only one question on CAM visits which prevents separate analysis based on frequency of visits. Furthermore, although the question mentioned several types of CAM practitioners, an even more comprehensive list would likely have increased the prevalence since it would enhance the respondents recall. The urban population was underrepresented in this study and a non-responder analysis of the 1997 data [18] found that older people were more likely not to answer the CAM question than younger people. Nevertheless, the age distribution was similar to other studies [6,19-21]. Chiropractors are authorised health personnel in Norway and were thus not included in the prevalence figures for CAM visits. This is likely to lower the prevalence compared to countries where chiropractors are CAM practitioners. Furthermore, CAM self care like use of products (herbs, vitamins) or practices (yoga) was not included and the prevalence is thus lower than for studies including such types of CAM. Studies which includes CAM self care [19], have similar profiles to this study.

The results may be affected by other factors that have not been recorded in both health surveys, i.e. the results may be affected by residual confounding. However, to our understanding, the main variables that lead to a visit to a CAM practitioner were included (general health, specific conditions, chronic conditions, socioeconomic situation). Importantly, the analyses were adjusted for having previously visited a general practitioner and chiropractor which in parts takes account of those who more frequently seek help as well as changes in health care utilisation between both surveys.

There was a substantial increase in the prevalence of CAM visits for both males and females during the 11 years, from 9.4% to 12.6%. This coincides with a general increase in visits to physicians in the same time period, 17% points for males and 11% points for females (calculated from the numbers in table 2). The observed prevalence in this study is close to half of the CAM visits, which included chiropractors, in Australia [6], where there was an increase from 20.3% in 1993 to 26.5% in 2004. It was similar to a smaller Israeli study where the prevalence of CAM visitors also including chiropractors increased from 6.1% in 1993 via 9.8% in 2000 to 12.4% in 2007 [7]. Since the prevalence of visitors to CAM practitioners excluding chiropractors was similar in Norway and the USA in 2002 [5] and since the prevalence for practitioner based therapies like acupuncture has increased in USA [11], the prevalence seems to be is similar in the northern hemisphere but considerably lower than in Australia. However, in all countries there has been an increase in practitioner based CAM use during the last decade.

Consistent findings in studies of CAM use have been that middle aged people are the highest users. In this study, the age group among male CAM visitors increasing most was those under 30 years. Also for females the younger age groups had the largest increase. This trend is also observed in the USA were the middle aged do not longer stand out as clearly when 2007 [11] is compared to 2002 [19], a situation similar to Ireland [10]. This indicates some fundamental changes starting to happen in CAM consumption. The reduced influence of psychiatric complaints in this study could point in the same direction, and in the USA it is also observed that there is a reduction in how frequent psychological problems like anxiety and depression are named as a reason for CAM use [11,19]. Since having a recent complaint was among the variables with the largest increase from 1997 to 2008 this further strengthens the assumption. It indicates that a larger proportion of the more healthy part of the population is increasing their visits to CAM practitioners. Although this study can give no answers to why this is so, one speculation might be that children who have been taken to a CAM practitioner by their parents have continued to use CAM. This speculation builds on the observed fact that in Norway, there was an increase in the proportion of children among patients visiting homeopaths, from one in ten in 1985 to one in four in 1998 [22].

## Conclusions

There has been an increase in the number of male and female adults who visit CAM practitioners from 1997 to 2008. The most prominent changes were that younger people of both genders with more limited complaints increased among the visitors.

## Competing interests

The authors declare that they have no competing interests.

## Authors' contributions

AS prepared the data file, performed the statistical analysis and drafted the manuscript. MBR and RJ helped to discuss the analysis and draft the manuscript. All authors have read and approved the final manuscript.

## Pre-publication history

The pre-publication history for this paper can be accessed here:

http://www.biomedcentral.com/1472-6882/11/61/prepub
